# Preliminary ultrasonography study of the pancreas in the dromedary camel (*Camelus dromedarius*)

**DOI:** 10.3389/fvets.2025.1495606

**Published:** 2025-02-05

**Authors:** El Hassania Lakhel, Khalid El Allali, Mohamed Rachid Achaâban, Rahma Azrib

**Affiliations:** ^1^Comparative Anatomy Unit, Department of Veterinary Biological and Pharmaceutical Sciences, Hassan IInd Agronomy and Veterinary Medicine Institute, Rabat, Morocco; ^2^Equine and Canine Unit, Department of Reproduction Medicine and Surgery, Agronomy and Veterinary Institute, Rabat, Morocco

**Keywords:** dromedary, camel, pancreas, anatomy, ultrasonography, reference patterns

## Abstract

**Introduction:**

Pancreatic lesions in camels can lead to significant economic losses. They are practically undetectable, as clinical signs alone are insufficient for specific diagnosis. Ultrasonography is a valuable diagnostic tool for evaluating the pancreas. However, ultrasonographic reference patterns of the pancreas in the camel are yet to be established. This study aimed to define the ultrasonographic appearance, reference values, location, and acoustic window for evaluating the pancreas in healthy camels.

**Methods:**

Eight adult and 14 young Moroccan camels were investigated by ultrasonography using a micro-convex probe with SIUI CTS-900 V and Samsung MH70A Doppler ultrasound scanners at 3.5 MHz.

**Results:**

The body of the pancreas was scanned just below the right kidney, behind the 12th rib; an ultrasonographic pattern of pancreatic parenchyma appeared as a hyperechoic elongated band, including the portal vein, with a Doppler flow response. The average thickness of the body was 3.60 ± 0.24 cm (*n* = 14) in young camels significantly lower than in adult camels 4.61 ± 0.26 cm (*n* = 8). The right lobe was scanned on the right side, adjacent to the duodenal ampulla and abomasum, beneath the liver along the 11th, 10th, and 9th intercostal spaces. The ultrasonographic pattern of parenchyma appeared as a hyperechoic triangle compared to the liver, including portal and duodenal-pancreatic veins, showing a Doppler flow response. The corresponding parenchyma thickness within the three intercostal spaces was 3.93 ± 0.33 cm, 4.40 ± 0.20 cm, and 3.46 ± 0.39 cm in the young camels (n = 14), and 4.99 ± 0.46 cm, 5.90 ± 0.27 cm, and 4.11 ± 0.68 cm in the adults (n = 8), respectively. The pancreatic major duct was seen as an anechoic circle with a hyperechoic wall, with a maximum diameter of 1 cm, and the left lobe scanned beneath the cranial extremity of the spleen; its ultrasonographic pattern showed an irregular hypoechoic band with a mean thickness of 2.32 ± 0.32 cm (*n* = 14) in young camels and 3.08 ± 0.52 cm (*n* = 8) in the adults, including a small splenic vein.

**Conclusion:**

Ultrasonography combined with Doppler techniques provides valuable information on pancreatic health, blood flow, and tissue perfusion, aiding early detection of pancreatic diseases and, consequently, minimizing economic losses in camel husbandry.

## 1 Introduction

Camel husbandry is known to be economically important in desert environments. Indeed, this species shows special adaptive peculiarities to cope with harsh climate conditions at a lower cost than other species, especially cattle and small ruminants (1). In addition, camels constitute a source of meat and dairy products (2).

The dromedary camel is also involved in agricultural vocations, such as transporting crops and diverse tourist activities. However, camel husbandry suffers from significant economic losses, mainly due to parasites, viral, and other diseases (3).

The prevalence of various diseases in camels, as highlighted by Hegazy and Fahmy (4) in their atlas of camel diseases, underscores significant clinical implications. In particular, pancreatic lesions are often overlooked in these publications despite their clear visibility during post-mortem examinations in slaughterhouses. In live camels, however, identifying pancreatic abnormalities remains challenging, as these conditions typically occur without pathognomonic clinical symptoms. This absence of overt signs complicates early diagnosis and emphasizes the urgent need for enhanced diagnostic protocols to detect pancreatic lesions in living animals, ultimately improving herd health management.

In this context, ultrasonography as a diagnostic tool in veterinary medicine has gained prominence recently (5). This non-invasive technique is rapid, safe, informative, and well-tolerated by animals, making it particularly suitable for camel use. Ultrasonography has been applied to explore various parts of the body, including the genital tract (6) and the digestive tract (7, 8). In Morocco, abdominal ultrasound investigations in camels have been conducted as part of academic veterinary theses, further demonstrating the growing recognition of role of ultrasonography in enhancing camel health diagnostics. This evolution in diagnostic practices may facilitate earlier detection of pancreatic lesions and contribute to better overall herd health management.

However, the existing literature has not adequately addressed ultrasonography of the pancreas in camels. For the first time, this study aimed to perform an ultrasonographic investigation of the pancreas in young and adult healthy dromedary camels. This investigation seeks to provide veterinary practitioners with referential ultrasonographic images of this organ and detailed descriptions of the examination protocols used.

## 2 Materials and methods

### 2.1 Animals

This study was conducted on 22 healthy dromedary camels, comprising 14 young camels under 2 years of age (seven males and seven females) and eight adult camels (four males and four females) under 10 years old.

Young camels had an average weight of 330.7 ± 23.4 kg, issued from farms in the southern region of Agadir (Latitude: 30°3′60′′N; Longitude: 9°3′60′′W). They had unlimited access to grazing and received supplements made of straw and barley. The camels were destined for slaughtering at the Sidi Bibi abattoir (region of Agadir, Southern Morocco). They were selected because, according to veterinary inspectors, most young dromedaries sacrificed for human consumption rarely show lesions in their carcasses and organs, and, therefore, increases the likelihood of obtaining ultrasound patterns without anomalies.

The four adult male dromedaries, weighing 585 ± 11 kg, originated from the Kaouki region of Essaouira, Morocco (Latitude: 31°21′20″N; Longitude: 9°47′50″W). They were used by nomads for tourism purposes and were clinically healthy. They have unrestricted access to grazing and receive straw and barley-based supplements.

The four adult female camels had an average weight of 575 ± 20 kg, were clinically healthy and kept in the sheepfold of the Comparative Anatomy Unit at the Hassan II Agronomy and Veterinary Medicine Institute in Rabat, Morocco (Latitude: 34.00492°N; Longitude: −6.8553°W). They were fed a special camelid industrial diet (Maraa, Alf Sahel, Morocco) and offered sufficient quantities of straw.

All experimental protocols were approved by the local ethics committee for animal science and health and veterinary public health (CESASPV) of the Hassan II Agronomic and Veterinary Medicine Institute, BP 6202, Rabat-Institutes, Morocco (ethical authorization number: CESASPV_2024_A17).

### 2.2 Methods

All the 22 clinically healthy camels, including 14 young camels (seven females and seven males), four adult females, and four adult males were assessed for the liver, kidney, and pancreatic functions to ensure their biochemical healthy statute. Blood samples were collected from all animals to measure urea, creatinine, AST, ALT, and amylase parameters. The results confirmed that all animals were within normal reference values. Following this confirmation, ultrasound examinations of the pancreas were performed. Young camels and adult males were examined using the SIUI CTS-900 V ultrasound scanner, while adult females underwent additional imaging with the Samsung MH70A Doppler ultrasound scanner.

The young camels (*n* = 14) were slaughtered for public consumption of the meat. They had previously been utilized for ultrasonographic examinations. A comprehensive examination of the carcasses was subsequently conducted, which revealed no abnormalities. Following this examination, a gross anatomical study of the pancreas was performed on these young camels.

All examinations of blood tests were conducted on a Mindray machine, using different kits for each parameter. Urea analyses were performed with urea kits that employed the urease-glutamate dehydrogenase method (UV). Creatinine levels were analyzed using a creatinine kit that utilized the sarcosine oxidase method. AST was measured with an aspartate amino-transferase kit (UV assay) following IFCC (International Federation of Clinical Chemistry and Laboratory Medicine) guidelines without pyridoxal phosphate activation. Similarly, ALT was assessed using an alanine amino-transferase kit (UV assay) according to IFCC standards, also without pyridoxal phosphate activation.

### 2.3 Gross anatomical study

**Figure 1:**
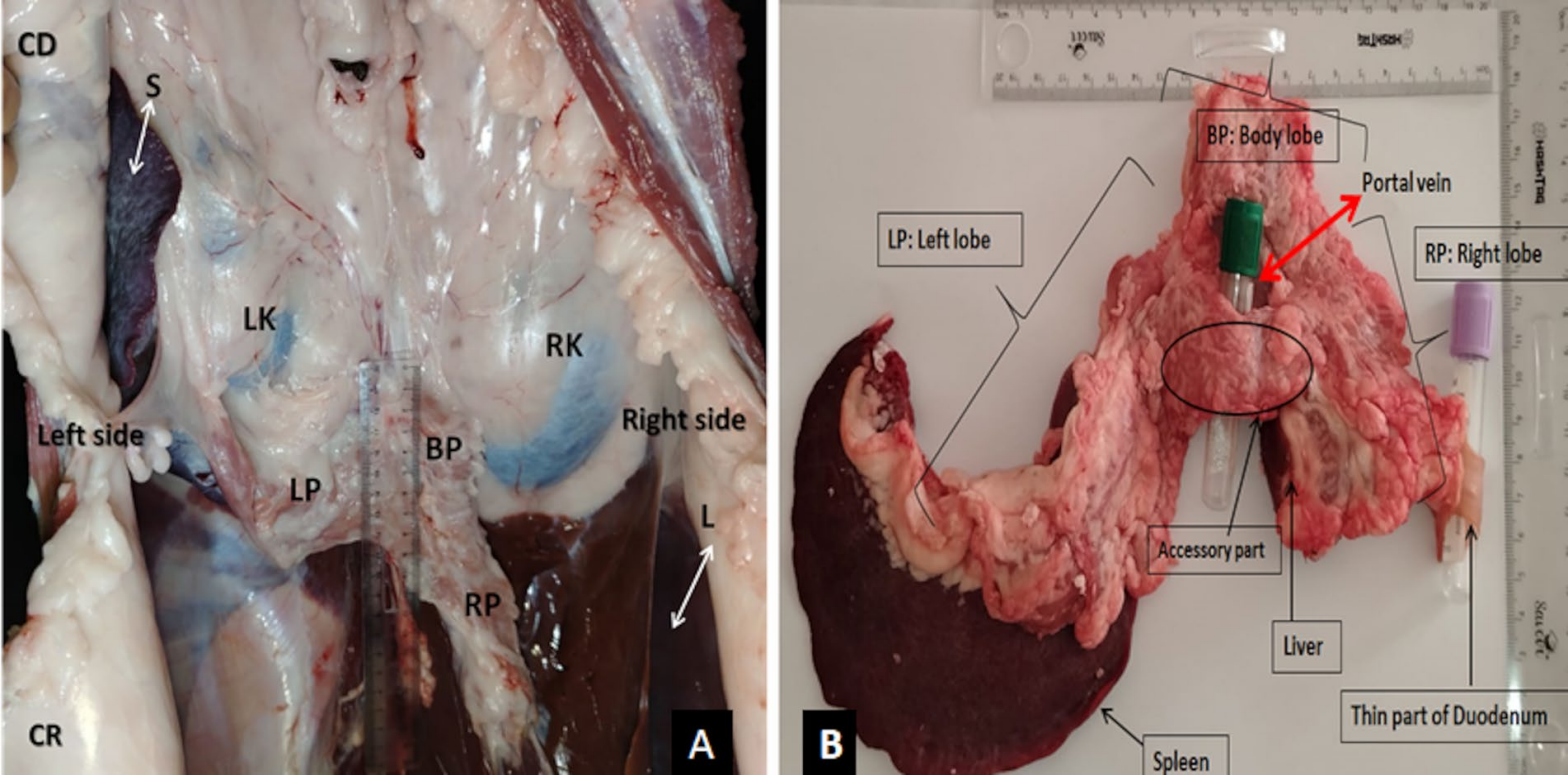
**(A)** Topography of the pancreas on the carcass of slaughtered camel (ventral view); **(B)** macroscopic conformation of the pancreas of the dromedary camel (dorsal view). RK, Right kidney; LK, left kidney; L, liver; S, spleen; LP, the left lobe of the pancreas; RP, the right lobe of pancreas; BP,: body lobe of the pancreas; CR, cranial; CD, caudal.

The gross anatomical study was realized on the pancreas of the young camels (*n* = 14) and was conducted after the camels were slaughtered. The objective was to explore the external conformation, topography, and relationships with neighboring organs (Figures 1A,B), which may help in interpreting ultrasound images. Subsequently, specific attention was given to the pancreas, which was anatomically studied to reveal its major macroscopic characteristics. The pancreas was carefully extracted to ensure its integrity, and the surrounding adipose tissue was removed to optimize the visualization of all pancreatic components and the external conformation (Figure 1B). Macroscopic measurements (width, length, and thickness) and the weight of the organ were also obtained.

### 2.4 Ultrasound examination

The ultrasound examinations were performed using an SIUI CTS-900 V ultrasound scanner in B-mode (brightness mode), a common imaging technique that provides two-dimensional images of internal structures (9), allowing for a detailed assessment of the pancreas.

Four adult female camels underwent two separate ultrasonography sessions, using both the SIUI CTS-900 V ultrasound scanner and the Samsung MH70A Doppler ultrasound scanner in order to distinguish vascular structures from other anatomical components using color flow. This dual approach aimed to enhance the visualization and assessment of vascular structures.

Despite the presence of experienced restraint technicians, an adult female camel exhibited excessive agitation during the ultrasound examinations, highlighting the challenges of performing such procedures on large and adult animals. Consequently, sedation has been used via intravenous injection of xylazine (Rompun^®^ 2%) at a dose of 0.25–0.5 mg per kg body weight. The same restraint method was applied during the injection, and the ultrasound examination was performed as part of the standard handling procedures for equines.

A 12-h restricted diet was not absolute but necessary to obtain superior quality images. Each animal was examined on an area of both sides, extending from the angle of the hip to the fourth rib dorsally and from the xiphoid to the stifle ventrally. All this area was correctly shaved. After generously applying transmission gel to the probe and the entire sheared area, ultrasonographic exploration was performed using a 3.5–7-MHz micro-convex and convex probe. Machine gain, depth, and frequency parameters were adjusted throughout the examination to ensure high-quality images.

**Figure 2:**
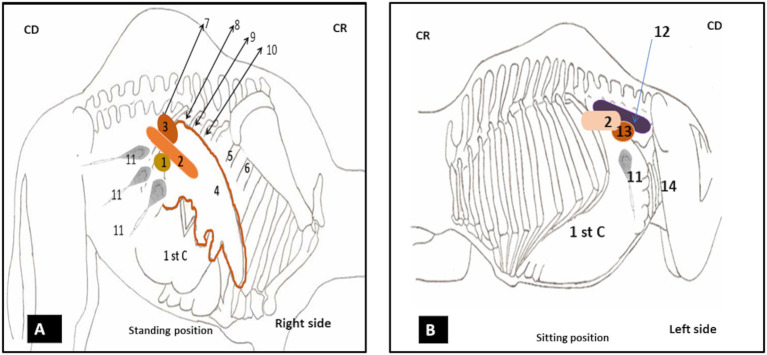
**(A)** Illustration of the positions of the probe for ultrasonography of the pancreas on the right side at a standing position. **(B)** Illustration of the position of the probe for ultrasonography of the pancreas on the left side at sitting position. 1: duodenal ampulla; 2: pancreas; 3: right kidney; 4: liver; 5: the 7th intercostal space; 6: the 6th intercostal space; 7: below the 11th intercostal space; 8: the 11th intercostal space; 9: the 10th intercostal space; 10: the 9th intercostal space; 11: probe; 12: spleen; 13: left kidney; 14: spiral colon; C1: first stomach compartment.

On the right side, the pancreatic body was scanned by positioning the probe behind the 12th rib in the para-lumbar fossa, directed cranially (Figure 2A), allowing for multiple scans; while, ultrasonography of the right lobe of the pancreas was performed using three acoustic windows corresponding to the last three intercostal spaces 11th, 10th, and 9th. Each space was examined from top to bottom, relying on the anatomical references, specifically by locating the liver parenchyma.

On the left side, the left lobe was scanned by positioning the probe below the last transverse processes of the lumbar vertebrae; ventrally to the spleen (Figure 2B).

This careful approach facilitated a thorough evaluation of all lobes of the pancreas and their anatomical relationships.

Measurements performed during ultrasonography examination included the thickness of different parts of the pancreas (body and lobes), the diameter of the portal vein on longitudinal and transversal scans, and the diameter of the pancreatic duct. All measurements were presented as mean ± standard deviation (SD), and the statistical analysis was performed using SPSS version 25.0 (2017; SPSS, Armonk, NY, United States).

A two-way analysis of variance (ANOVA), followed by the Holm–Sidak *post-hoc* test, was used to evaluate the existence of significant differences in ultrasonographic measurements between adult and young and between females and males categories. A *p*-value of ≤0.05 was deemed to be statistically significant for all tests conducted.

## 3 Results

### 3.1 Gross anatomy of the pancreas

The pancreas in camels is characterized by the development of its left lobe and a relatively reduced right lobe. It generally consists of a body, two lobes, and an accessory part; with an average weight of 124.5 ± 49.1 g (see Figure 1B). The pancreas exhibits similar shape and size in both females and males.

The pancreatic body (*Corpus pancreatic*) in camels is located below the first and second lumbar vertebrae. It is in contact with the portal vein, the caudate hepatic lobe, and the diaphragm. It is also related to the transverse colon and kidney. The width, length, and thickness are approximately 4.8 ± 0.5 cm, 7.6 ± 1.1 cm, and 1.5 ± 0.2 cm, respectively.

The right pancreatic lobe (*Lobus pancreatis dexter*) is prismatic in shape and projects backward and to the right. It is related to the second duodenal flexure and is contained in the meso-duodenum. The width, length, and thickness average 2.1 ± 0.2 cm, 6.9 ± 0.4 cm, and 0.7 ± 0.1 cm, respectively.

The left pancreatic lobe *(Lobus pancreatis sinister)* is larger than the right lobe and is positioned obliquely between the folds of the greater omentum. Anatomically, it is associated with several structures, including the first stomach compartment (rumen), transverse colon, descending colon, spleen, left kidney, and adrenal gland. The average dimensions of the left lobe are as follows: a width of 2.9 ± 1.1 cm, a length of 21.7 ± 7.5 cm, and a thickness of 1 ± 0.3 cm.

The accessory part *(Processus uncinatus)* unites the right and left lobes, forming an annular pancreas around the portal vein; the width, length, and thickness are, on average, 2.5 ± 0.4 cm, 1.3 ± 0.2 cm, and 0.4 ± 0.1 cm, respectively.

### 3.2 Animal examination and laboratory findings

All the examined camels were clinically healthy, showing no signs of pathology. Vital parameters, such as body temperature, respiratory rate, heart rate, and rumination, were within normal limits, indicating good overall health. The blood parameters analyses conducted on the 22 camels to assess kidney function revealed levels of 2.0 ± 0.6 mg/100 mL for urea and 1.04 ± 0.3 mg/100 mL for creatinine, both of which were within normal limits. These values were consistent with the reference ranges established by Bengoumi (10), indicating urea levels between 2 and 4 mg/100 mL and creatinine levels below 2 mg/100 mL.

Additionally, parameters assessing liver function showed levels of 93.4 ± 16.2 u/l for AST and 18.27 ± 4.4 u/l for ALT, both of which were within normal limits. These values were consistent with the reference ranges established by Bengoumi (10), both being lower than the thresholds of 115u/l for AST and 24u/l for ALT.

Finally, the measured levels of amylase activity 854.74 ± 321.27 u/l were within the normal range but lower than the threshold of 2,325 u/l as reported by Mura et al. (11).

### 3.3 Ultrasonographic appearance of the pancreas

The ultrasonographic exploration of the pancreas was performed on a total of 22 camels using a SIUI CTS-900 V ultrasound scanner equipped with micro-convex and convex probes (3.5–7 MHz). Multiple trials showed that the micro-convex probe at a frequency of 3.5 MHz yielded the highest quality images, despite the complex anatomy of the pancreas and limited access in the intercostal spaces.

Following the anatomical and morphological structure of the pancreas, which is composed of three parts (the body, the right lobe, and the left lobe; see Figure 1B), the ultrasonographic examination was performed using different acoustic windows (Figure 2) for exploration, encompassing all three components.

On four adult female camels, ultrasonography was performed using both the SIUICTS-900 V ultrasound scanner and the Samsung MH70A Doppler ultrasound scanner at 3.5 MHz to assess vascularization. The results confirmed active vascularization observed with the macroscopic examination, identifying the portal vein and pancreatico-duodenal vein among the observed vessels.

**Figure 3:**
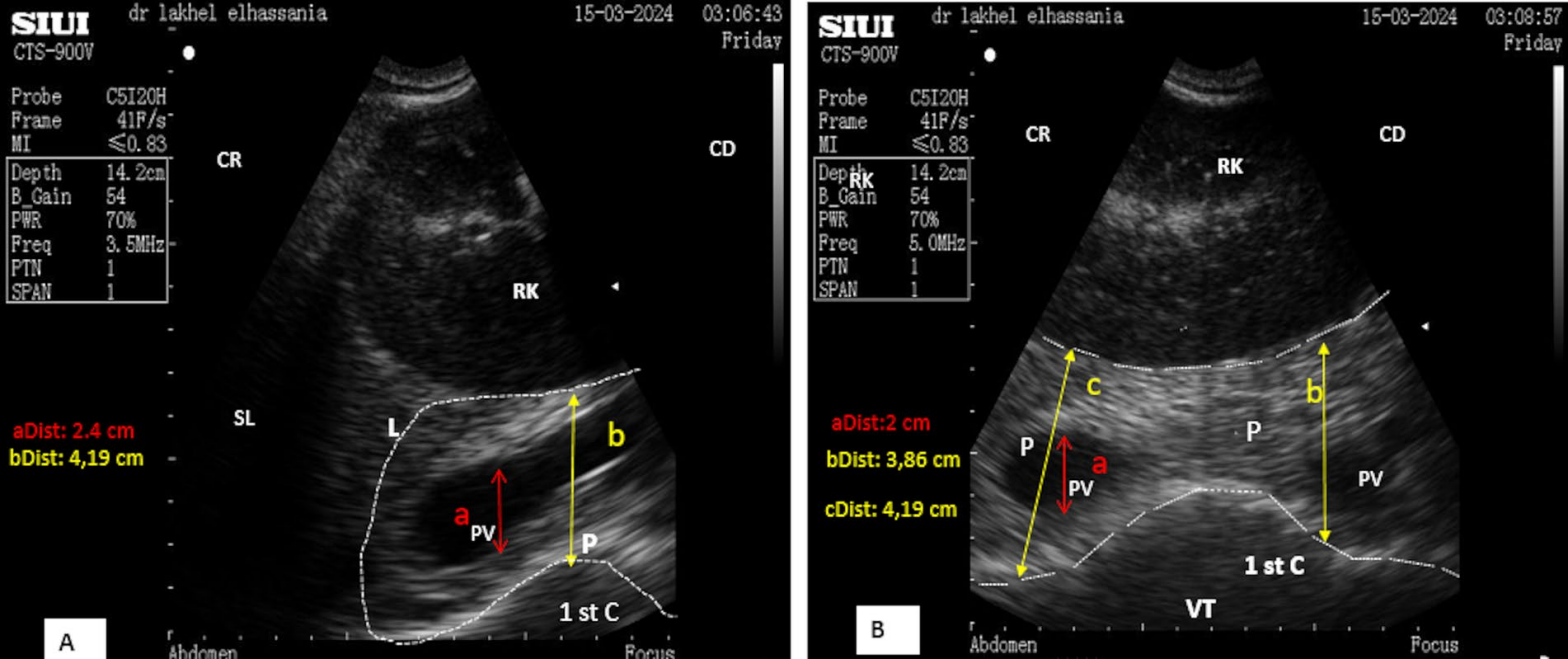
**(A)** Longitudinal scan of the body lobe of the pancreas caudally to the upper third of the 12th rib. **(B)** Transverse scan of the body lobe of the pancreas caudally to the upper third of the 12th rib. RK, Right kidney; PV, portal vein; 1st C, first stomach compartment; P, pancreas; L, liver; SL, shadow of the lung; VT, ventral; (aDist), diameter of portal vein; (bDist) and (cDist), thickness of pancreatic body; CR, cranial; CD, caudal; VT, ventral.

The ultrasonography of the pancreatic body was performed on the right flank by positioning the micro-convex probe, just below the transverse processes of the first and second lumbar vertebrae behind the upper third 12th rib. The right kidney was initially identified and localized, and then, the probe was directed cranio-ventrally toward it. The ultrasound frequency was set to 3.5 MHz, with a depth of 14.2 cm and a gain of approximately 50%. The ultrasonographic pattern of pancreatic parenchyma of the body appears hyperechoic, elongated, and irregular in shape, located closely to the anterior visceral surface of the kidney, caudal to the ventral surface of the liver (Figure 3A). The average thickness observed was 3.58 ± 0.22 cm in longitudinal scans of young male camels (Figure 3B), which is quite similar to the 3.63 ± 0.28 cm found in young female camels. However, this thickness was significantly higher (*p* < 0, 0001) in adults than in young camels, 4.64 ± 0.31 cm (*n* = 4), and 4.58 ± 0.24 cm (*n* = 4), respectively, for adult camels males and females. No significant differences were observed between males and females within young and adult categories.

The average thickness of the body was 3.60 ± 0.24 cm (*n* = 14) in young camels, significantly lower than in adult camels 4.61 ± 0.26 cm (*n* = 8).

Furthermore, the position of the animal may affect the pancreas, whereas the thickness remained consistent across standing and sitting positions as well as transverse and longitudinal scans. In the sitting position, the body lobe of the pancreas was identified via ultrasonography in the upper third of the 12th rib. In contrast, in the standing position, it was located behind the middle third of the 12th rib.

The pancreatic parenchyma of the body contains a single large vessel running through it, representing the portal vein, which exhibits a clear color flow Doppler. In longitudinal scans, this vessel appears as an anechoic channel (Figure 3A), with a mean diameter of 1.83 ± 0.19 cm (*n* = 14) in young camels, statistically lower than that observed in adult camels 3.08 ± 0.15 cm (*n* = 8). In transverse scans, it appears as an anechoic circle (Figure 3B). Similar significant differences in the diameter of the portal vein were observed between young and adult camels on a transversal scan 1.80 ± 0.19 cm (*n* = 14) vs. 3.14 ± 0.18 cm (*n* = 8), respectively.

Ultrasonography of the right pancreatic lobe was performed through three distinct acoustic windows to ensure optimal visualization, guided by anatomical reference points.

**Figure 4:**
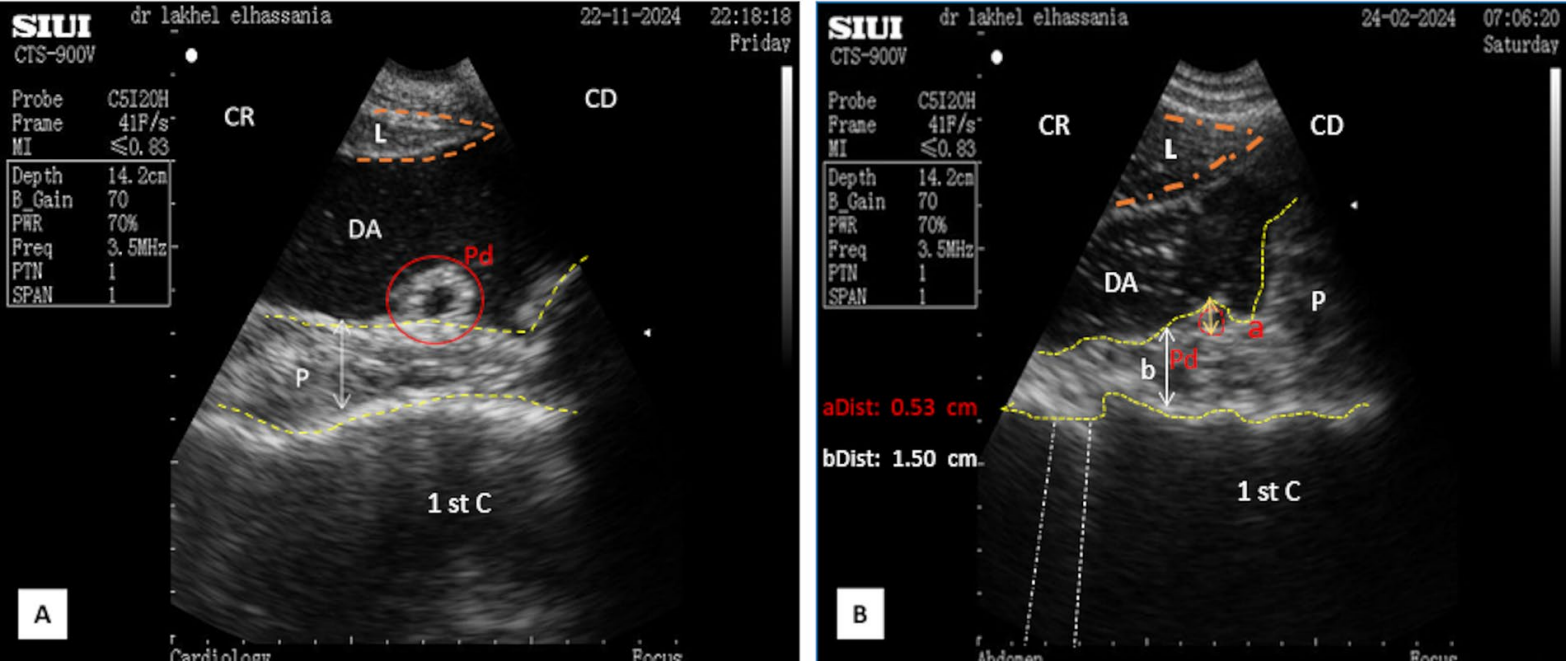
**(A)** Transverse scan of the right lobe of the pancreas in the middle third of the 11th intercostal space. **(B)** Identification of structures on the ultrasound image. 1st C, (first stomach compartment); P, pancreas (yellow dotted outline); L, liver (orange dotted outline); DA, duodenal ampulla; Pd, pancreatic duct (red circle); (aDist), diameter of pancreatic duct; (aDist), thickness of the right lobe of the pancreas; CR, cranial; CD, caudal.

*The first acoustic window, located at the 11th intercostal space*, identified the proximal margin of the liver from the midline due to the pancreas proximal to the liver. This margin was found to be, on average, 45.25 ± 6.4 cm in adult camels and 39.42 ± 3.2 cm in young camels, whereas no significant differences were found between males and females. The probe was oriented ventrally toward this margin, where the pancreatic parenchyma of the right lobe appeared hyperechoic compared to the liver. It is located caudo-ventrally, intercalated by the duodenal ampulla (Figures 4A,B).

**Figure 5:**
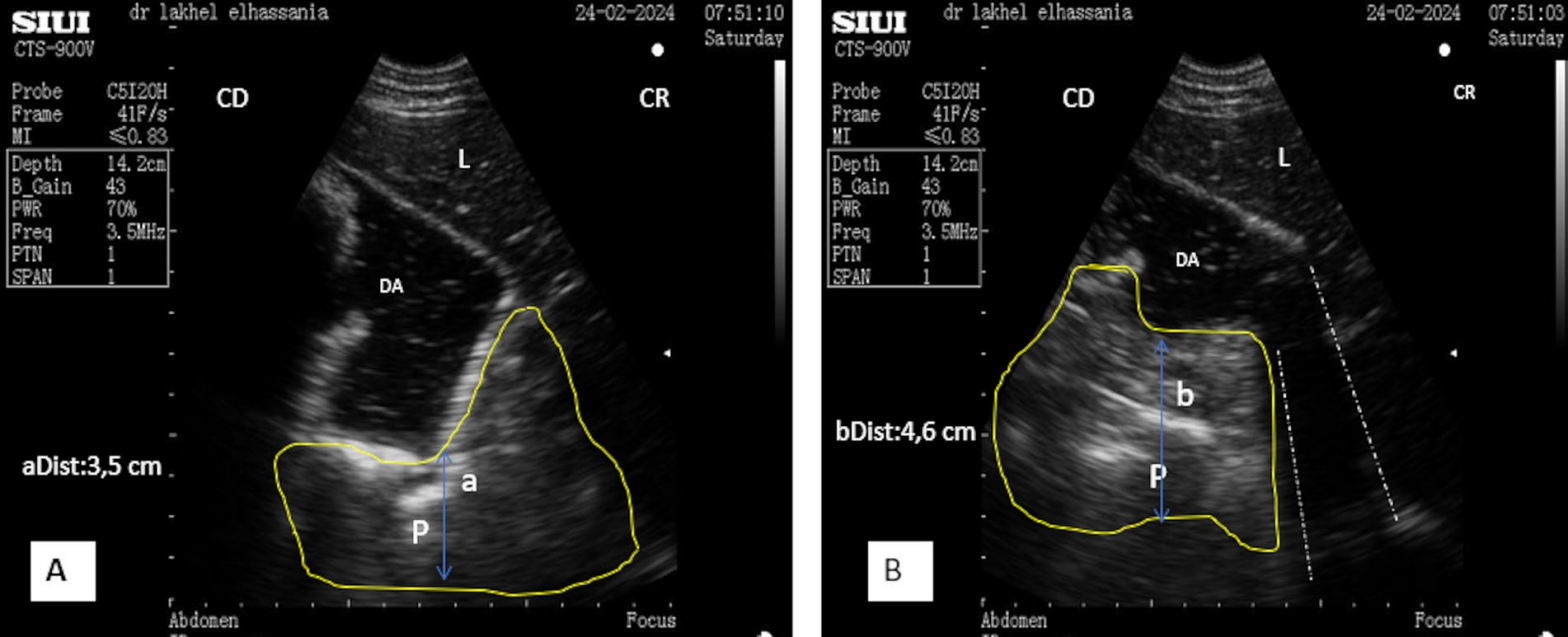
**(A)** Longitudinal scan of the right lobe of the pancreas in the middle of the 11th intercostal space in a resting phase. **(B)** Longitudinal scan of the right lobe of the pancreas in the middle of the 11th intercostal space in the contracting phase. L, liver; DA, duodenal ampulla; P, pancreas (yellow outline); (aDist), thickness of the right lobe in resting phase; (bDist), thickness of the right lobe of the pancreas in contraction phase; (White dotted line), acoustic shadow; CR, cranial; CD, caudal.

In the standing position, the pancreatic parenchyma was scanned starting from the middle third of the 11th intercostal space, whereas in the sitting position, it was scanned from the upper third, remaining closely adjacent to the duodenal ampulla. The thickness was slightly smaller in the sitting position, particularly when the duodenal ampulla was full (Figures 5A,B).

In the middle third of the 11th intercostal space, the ultrasonographic pattern of the parenchyma of the right lobe displayed an elongated, irregular band-shaped structure, particularly when the scan did not reveal the portal vein. The thickness of the pancreatic parenchyma of the right lobe was significantly smaller in young camels, measuring 1.61 ± 0.15 cm (*n* = 14), and 2.15 ± 0.24 cm (*n* = 8) in adult camels during transverse scans, regardless of sex (Figure 4B). Additionally, the major pancreatic duct was visible, with a mean width of 0.67 ± 0.05 cm (*n* = 14) for young camels and 0.79 ± 0.05 cm (*n* = 8) for adult camels. No statistical differences were observed for this parameter, regarding age and sex (Figure 4A). In contrast, when the portal vein was included in transverse scans, the pattern appeared triangular, with a thickness of 3.03 ± 0.35 cm (*n* = 14) for young camels and 4.77 ± 0.54 cm (*n* = 8) for adults. In longitudinal scans, the parenchyma appeared irregular and trapezoid-shaped measuring 3.93 ± 0.33 cm for young camels and 4.99 ± 0.43 cm for adults (Figures 5A,B).

**Figure 6:**
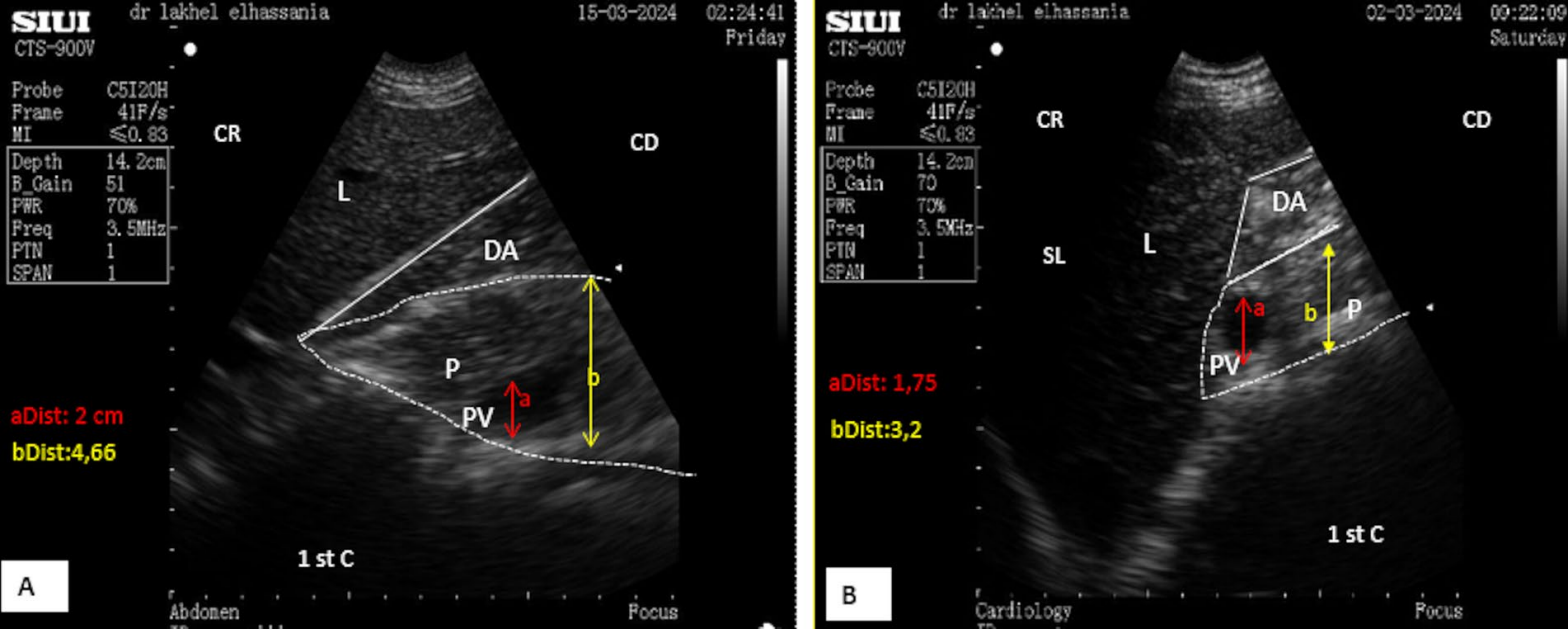
**(A)** Transverse scan of the right lobe of the pancreas in the middle of the 10th intercostal space. **(B)** Transverse scan of the right lobe of the pancreas in the upper third of the 10th intercostal space. PV, Portal vein; 1st C, (first stomach compartment); P, pancreas (white dotted outline); L, liver; DA, duodenal ampulla; SL, shadow of the lung; (aDist), diameter of portal vein; (bDist), thickness of pancreatic of the right lobe of the pancreas; CR, cranial; CD, caudal.

The portal vein and the duodenal-pancreatic vein appeared as anechoic channels in longitudinal scans and as anechoic circles in transverse scans, which was confirmed by color flow Doppler ultrasonography. The average diameter of the portal vein was 1.52 ± 0.12 cm for young camels and 3.12 ± 0.45 cm for adults, regardless of sex (Figure 6).

*In the second window, the scan starts from the upper to the lower area in the 10th intercostal space*, identifying the proximal margin of the liver from the dorsal midline at 48.12 ± 4.0 cm for adult camels and 44.14 ± 3.9 cm for young camels, with no significant differences regarding sex. In the standing position, the pancreatic parenchyma was scanned starting from the middle third of the 10th intercostal space, while in the sitting position, the right lobe was pushed upward, and therefore, it was scanned from the upper third.

**Figure 7:**
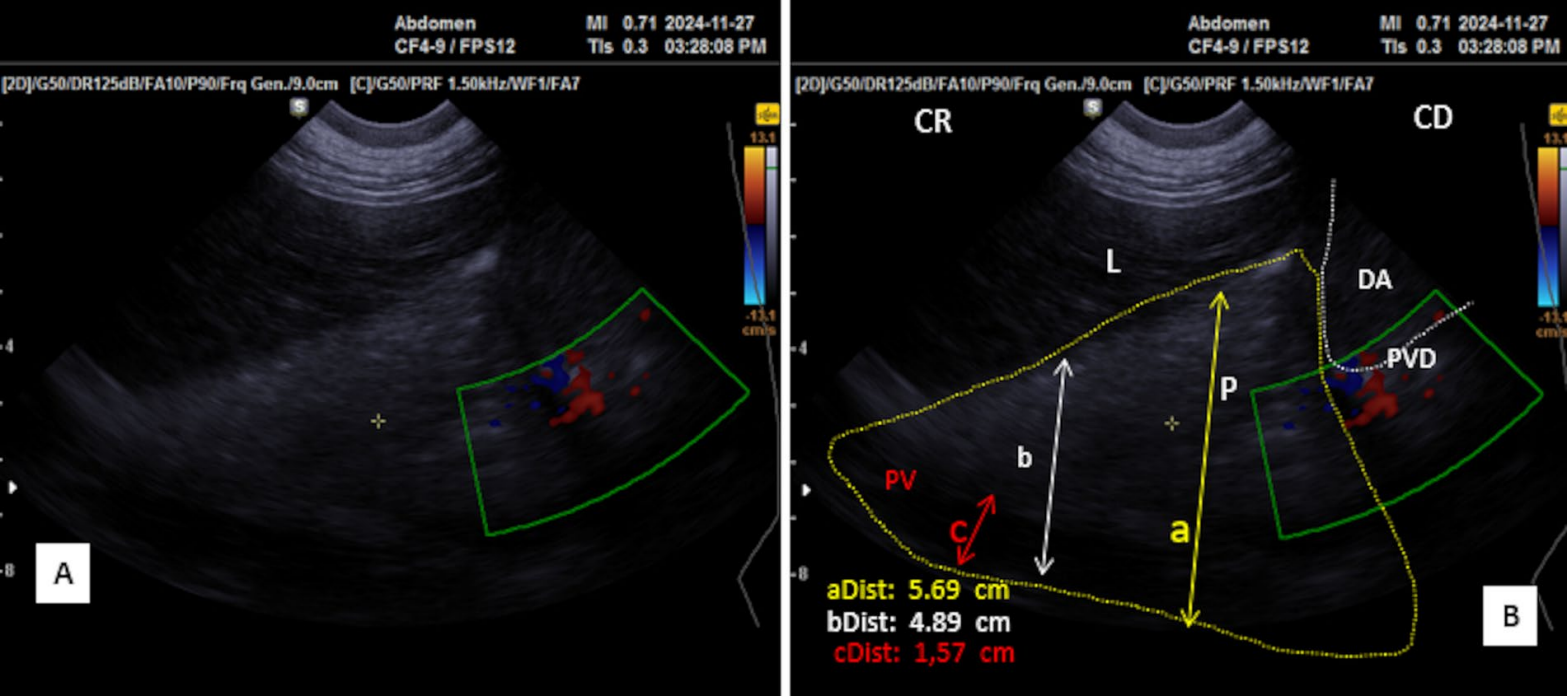
Transverse scan of the right lobe of the pancreas in the middle third of the 10th intercostal space **(A)**. **(B)** Identification of structures on the ultrasound image. P, pancreas (yellow dotted outline); L, liver; DA, duodenal ampulla (white dotted outline); PV, portal vein; PDV, pancreatico-duodenal vein; PD, pancreatic duct; (aDist) and (bDist), thickness of pancreatic of the right lobe of the pancreas; (cDist), diameter of portal vein; CR, cranial; CD, caudal.

**Figure 8:**
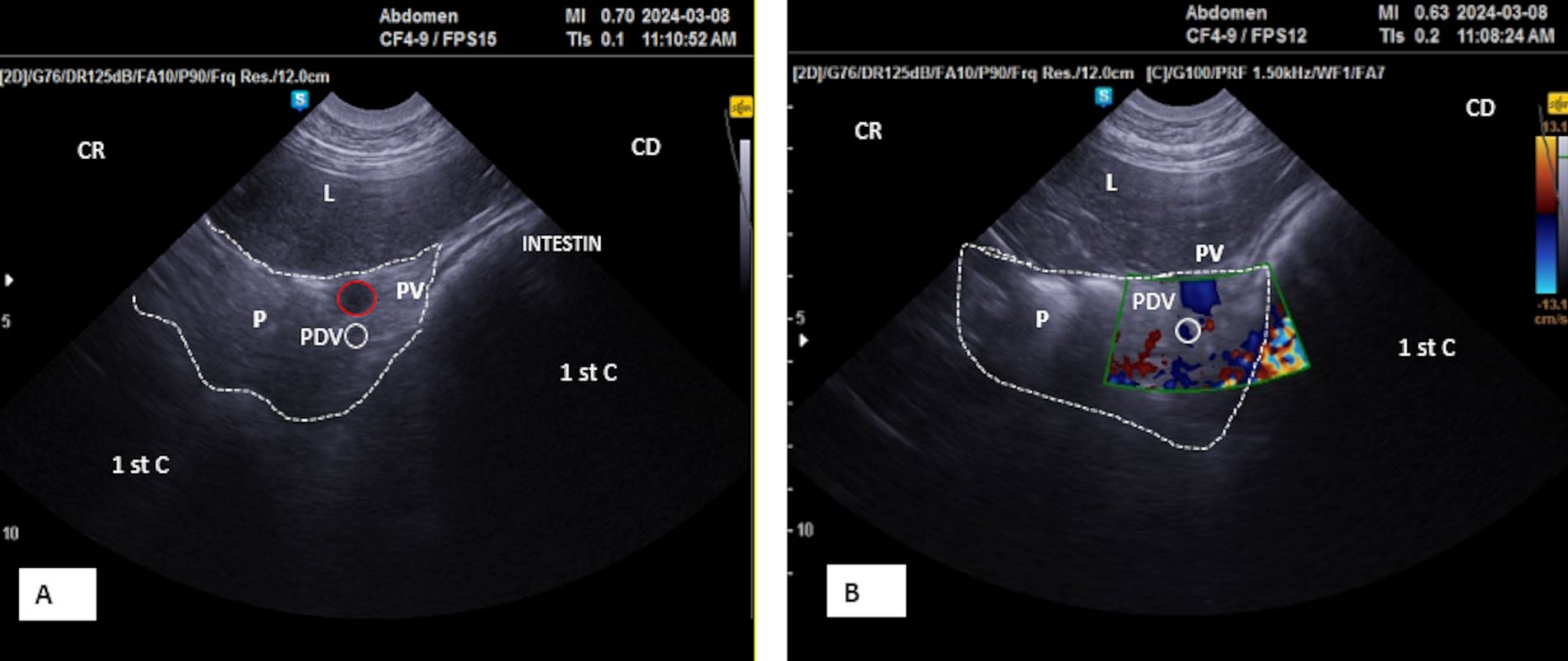
**(A)** Transverse scan of the right lobe of the pancreas of the middle third of the 10th. **(B)** Transverse scan of the right lobe of the pancreas of the middle third of the 10th using Doppler mode. P, Pancreas (white dotted outline); PV, portal vein (red circle); PDV, pancreatico-duodenal vein (white circle); L, liver; 1st C, (first stomach compartment); CR, cranial; CD, caudal.

After directing the probe ventrally toward this margin, it appeared hyperechoic compared to the liver, ventro-caudally to the duodenal ampulla. Its shape was triangular during the resting phase (Figure 6A) and took on a prism-like appearance during the contracting phase of digestion (Figure 6B), as observed when using both the SIUI CTS-900 V and Samsung MH70A ultrasound scanners (Figures 7, 8).

**Figure 9:**
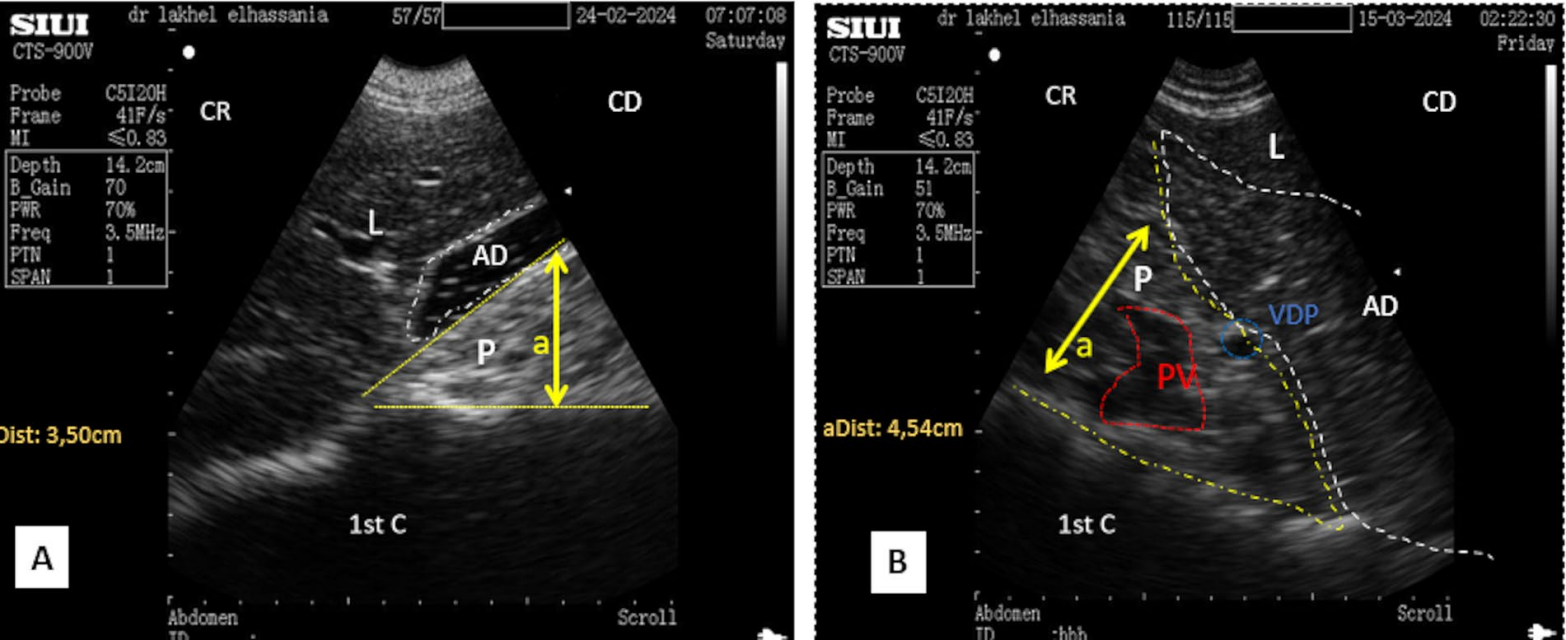
**(A)** Transverse scan of the right lobe of the pancreas in the middle third of the 10th intercostal space. **(B)** Transverse scan of the right lobe of the pancreas in the lower third of the 10th intercostal space; 1st C, (first stomach compartment); P, pancreas (yellow dotted outline); L, liver; AD, duodenal ampulla (white dotted outline); PV, portal vein (red dotted outline); (aDist), thickness of pancreatic of the right lobe of the pancreas; CR, cranial; CD, caudal.

Placing the probe in the middle third of the 10th intercostal space allowed an optimal view of the right lobe. The pancreatic parenchyma was wider than observed in previous positions, with the pancreatic duct visible and having a similar dimension as that observed in the 11th intercostal space (Figures 6, 9A). The average thickness of the parenchyma was 4.40 ± 0.20 cm (*n* = 14) for young camels and 5.90 ± 0.25 cm (*n* = 8) for adults. The portal vein was identified in the center (Figures 6A,B), along with the pancreatico-duodenal vein, which responded to color flow Doppler ultrasonography (Figures 8A,B). In the transversal scan, the portal vein diameter averaged 1.83 ± 0.14 cm (*n* = 14) in young camels and 3.23 ± 0.51 cm (*n* = 8) in the adults.

By moving the probe ventrally in the lower part of the 10th intercostal space, the pancreatic parenchyma of the right lobe, along with the portal vein and pancreatico-duodenal vein, was still observed, but with lesser thickness than previous probe positions (Figure 9B).

*In the third window, the scan starts from the upper to the lower area in the 9th intercostal* space, identifying the proximal margin of the liver from the dorsal midline at 52.1 ± 4.1 cm for adult camels and 47.6 ± 3.5 cm for young camels, with no significant differences regarding sex. However, in the standing position, the pancreatic parenchyma was scanned from the middle third of the 9th intercostal space, adjacent to the abomasum and the first compartment of the stomach (rumen). In the sitting position, it was scanned from the upper third.

The mean thickness of the pancreatic parenchyma in longitudinal scans averages 3.46 ± 0.39 cm (*n* = 14) in young camels and 4.11 ± 0.68 cm (*n* = 8) in the adults. This area includes only the portal vein, which shows a larger width: 1.67 ± 0.35 cm (*n* = 14) in young camels and 2.95 ± 0.18 cm (*n* = 8) in adults.

**Figure 10:**
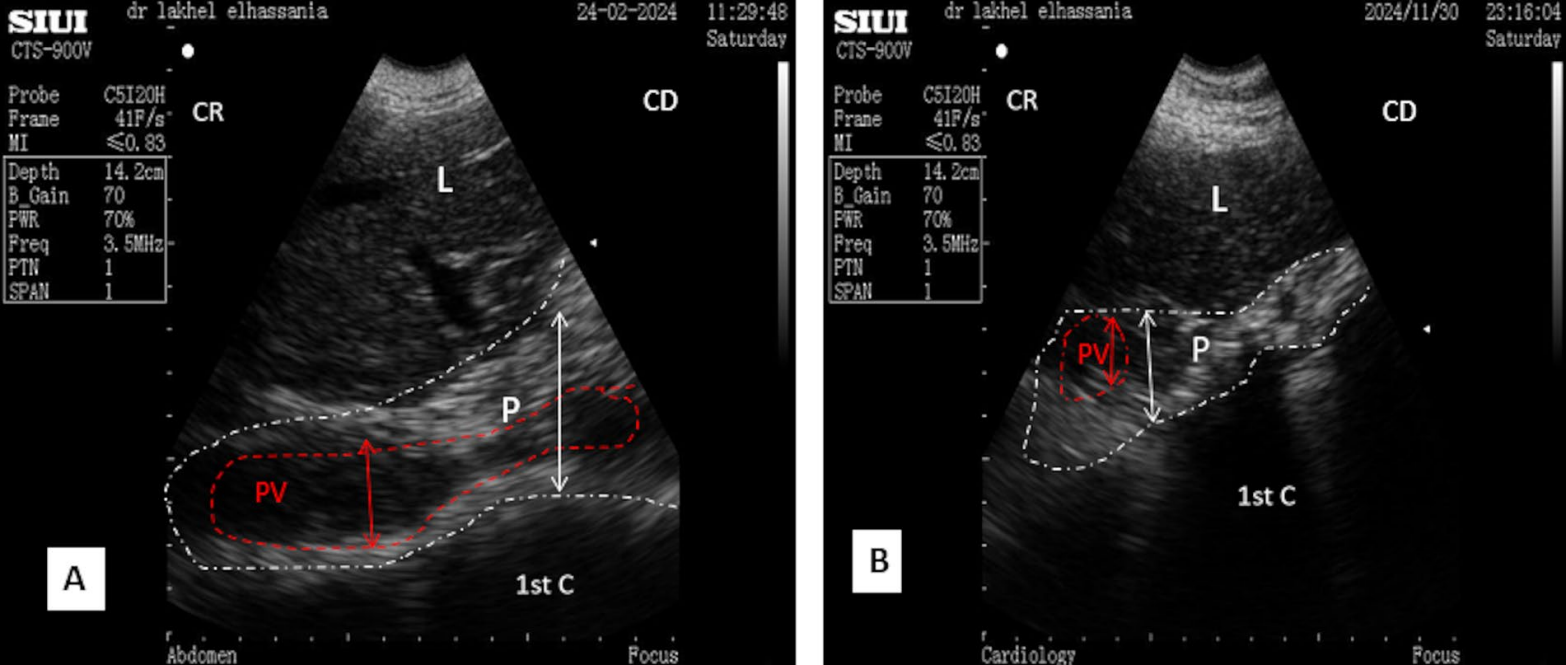
**(A)** Longitudinal scan of the right lobe of the pancreas in the middle third of the 9th intercostal space. **(B)** Transversal scan of the right lobe of the pancreas in the middle third of the 9th intercostal space; 1st C, (first stomach compartment); P, pancreas (white dotted outline); L, liver; PV, portal vein (red dotted outline); CR, cranial; CD, caudal.

In the upper third of the 9th intercostal space, the scan shows a triangular-shaped ultrasonographic pattern in the upper third of the 9th intercostal space, which becomes prism-like during contraction. However, in the middle third, it appears as an irregular shape (Figure 10A).

In contrast, the thickness is less in the lower third (Figure 10B), resembling a small band compared to previous spaces during the resting phase of ruminal contraction and in the sitting position.

**Figure 11:**
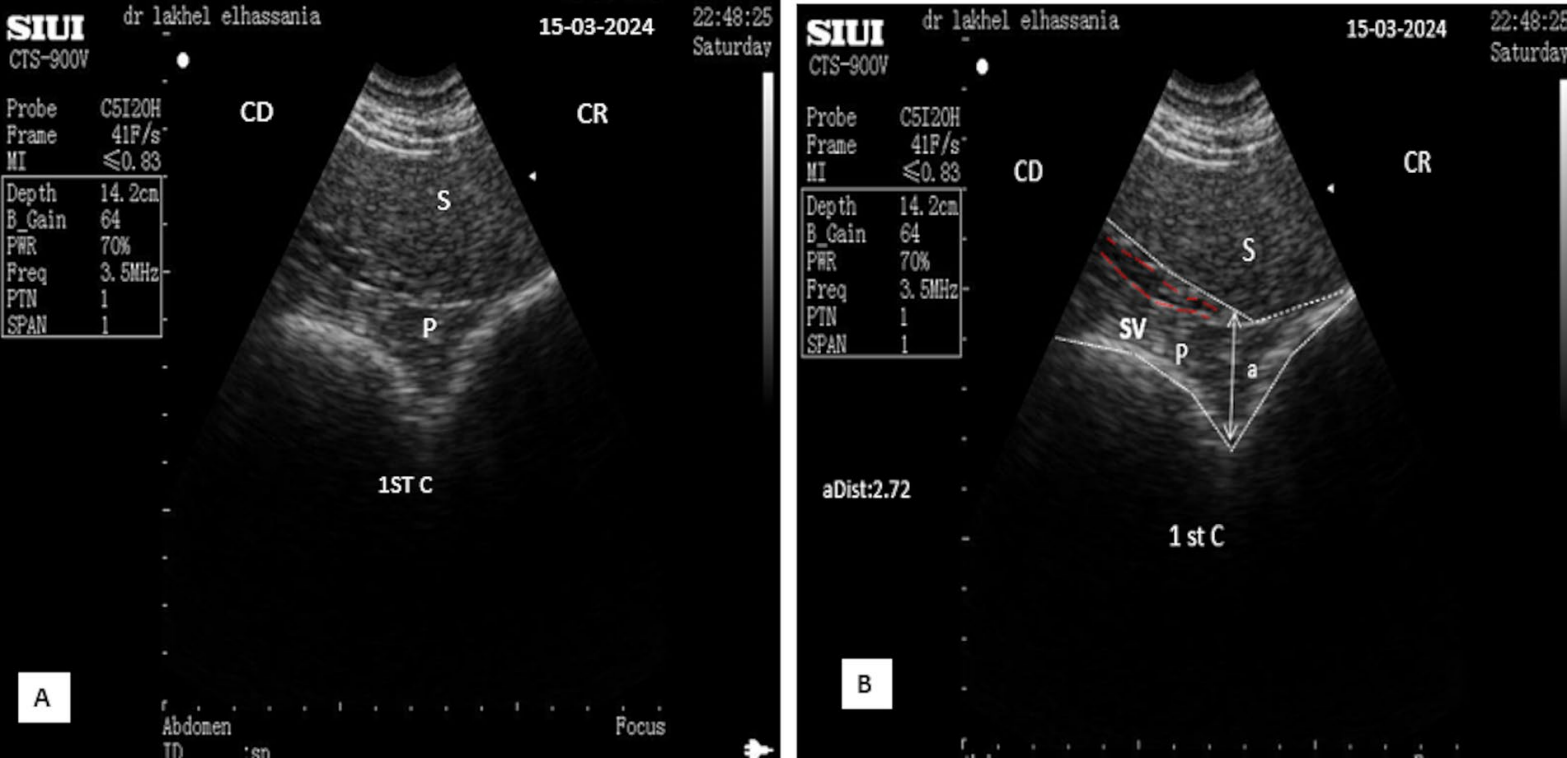
**(A)** Longitudinal scan of the left lobe of the pancreas ventrally to the anterior extremity of the spleen under 4th transverse process of the lumbar vertebra in the left flank. (**B**: Identification of structures on the ultrasound image). S, Spleen; P, pancreas (white dotted outline); SV, splenic vein (red dotted outline); (aDist), thickness of the left lobe of the pancreas; CR, cranial; CD, caudal.

Ultrasonography of the left lobe of the pancreas proved challenging. Based on anatomical and topographical references seen in slaughtered animals (Figure 1A); the probe was positioned in a region defined cranially by the last rib, caudally by the fifth transverse processes of the lumbar vertebra and the left kidney, ventrally by the first stomach compartment “rumen,” and dorsally by the spleen. The ultrasound frequency was also set to 3.5 MHz, with a depth of 14.2 cm and a gain of approximately 70%. After identifying the left kidney and the cranial part of the spleen, the probe was directed cranially and ventrally toward the anterior extremity of the spleen. The pancreatic parenchyma appeared as a small hyperechoic band compared to the kidney and slightly hypoechoic compared to the spleen, situated above the first stomach compartment (Figure 11). Because of its elongated form averaging 21.7 ± 7.5 cm in young camels, the left lobe, like the right lobe and body of the pancreas, cannot be fully visualized in a single scan and, therefore, multiple scans are necessary to the entire imaging of its caudal end. These scans should begin by placing the probe ventrally to the surface of the spleen and gradually moving it caudally until reaching the fifth lumbar vertebra.

**Figure 12:**
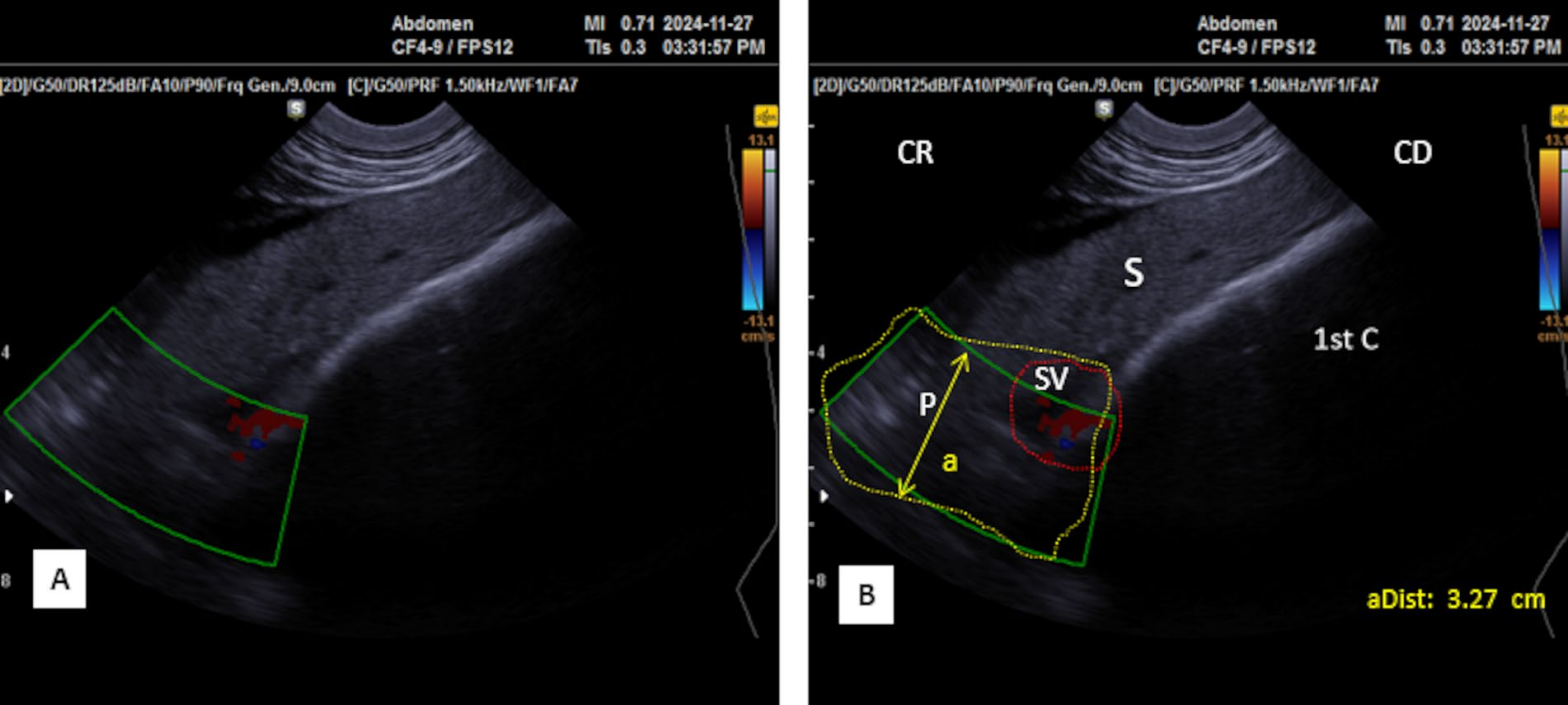
**(A)** Transversal scan of the left lobe of the pancreas ventrally to the anterior extremity of the spleen under third transverse process of the lumbar vertebra in the left flank Samsung MH70A Doppler ultrasound scanner. (**B**: Identification of structures on the ultrasound image). S, Spleen; P, pancreas (yellow outline); SV, splenic vein (red dotted circle); (aDist), thickness of the left lobe of the pancreas; 1st C, (first stomach compartment); CR, cranial; CD, caudal.

**Figure 13:**
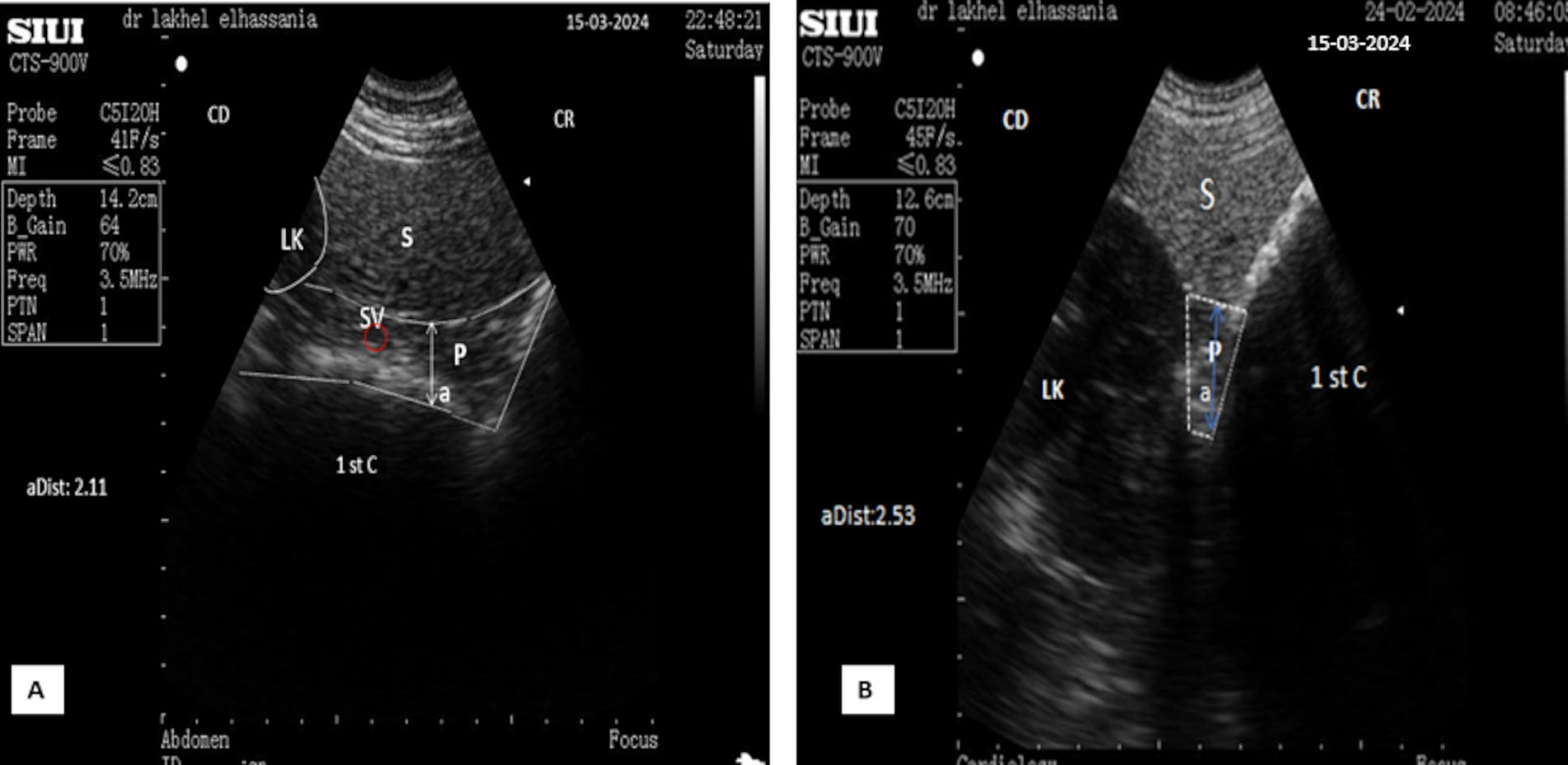
**(A)** Transverse scan of the left lobe of the pancreas under 3rd transverse process of the lumbar vertebra in the left flank. **(B)** Transverse scan ventrally to the anterior extremity of the spleen under 2^nd^ transverse process of the lumbar vertebra in the left flank. S, Spleen; Lk, left kidney; P, pancreas (white outline); SV, splenic vein (red dotted circle); (aDist), thickness of the left lobe of the pancreas; 1st C, (first stomach compartment); CR, cranial; CD, caudal.

The parenchyma of the left lobe has an average thickness of 2.32 ± 0.32 cm (*n* = 14) in young camels and significantly higher (*p* < 0.0001) in adult camels: 3.08 ± 0.52 cm (*n* = 8). The pancreatic parenchyma appears hyperechoic compared to the kidney and slightly hypoechoic compared to the spleen (Figure 11). The portal vein is absent in this area; however, the splenic vein is visible, when using Doppler color flow (Figure 12), and appears as a small anechoic channel on longitudinal scans (Figure 11) and as an anechoic circle on transversal scans (Figure 13).

Ultrasonography can be a challenge to delineate the accessory lobe. However, as it forms the pancreatic annulus around the portal vein, Doppler imaging helps define the vein and, therefore, can visualize the surrounding structures (Figure 8).

**Figure 14:**
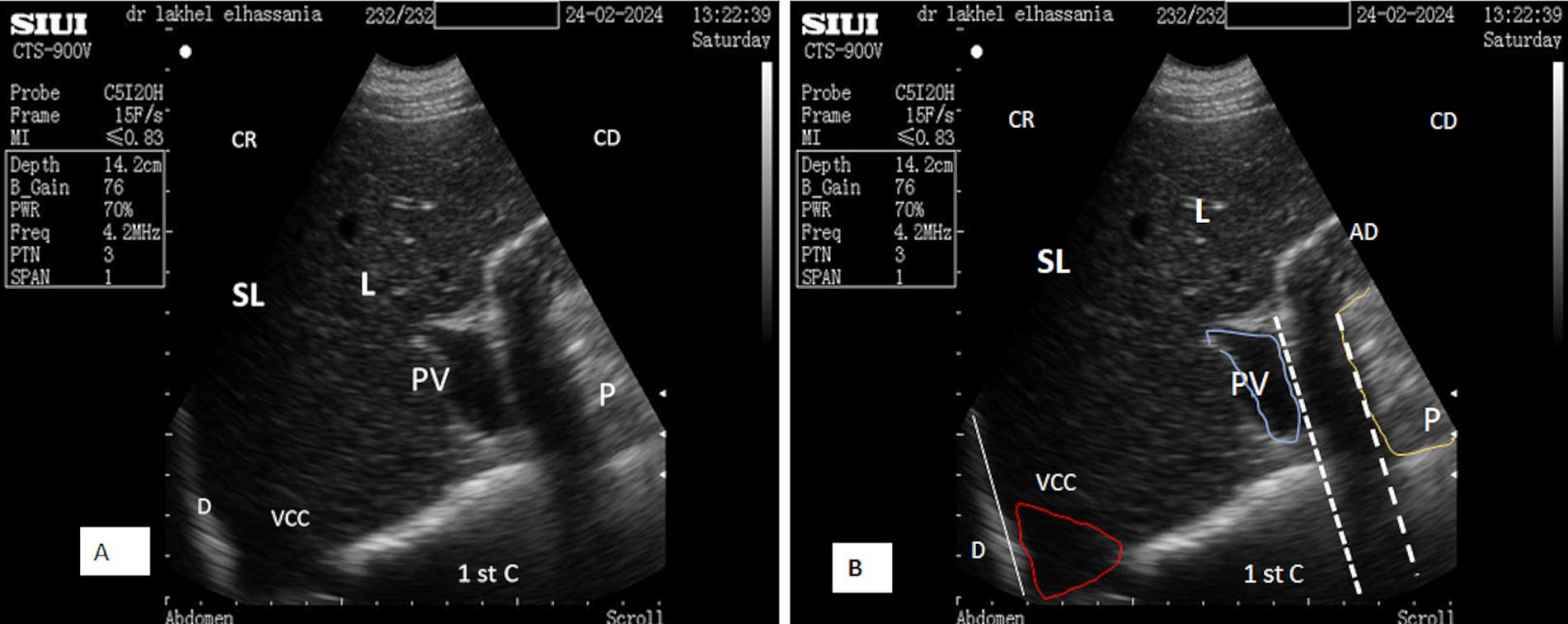
**(A)** Transverse scan of the right lobe of the pancreas in the upper third of the 10th intercostal space in the contraction phase. **(B)** Identification of structures on the ultrasound image. SL, shadow of the lung; L, liver; AD, duodenal ampulla; P, pancreas (yellow line); white dotted line, acoustic shadow; PV, portal vein (blue outline); VCC, caudal vena cava (red outline); D, diaphragm; 1st C, (first stomach compartment); CR, cranial; CD, caudal.

**Figure 15:**
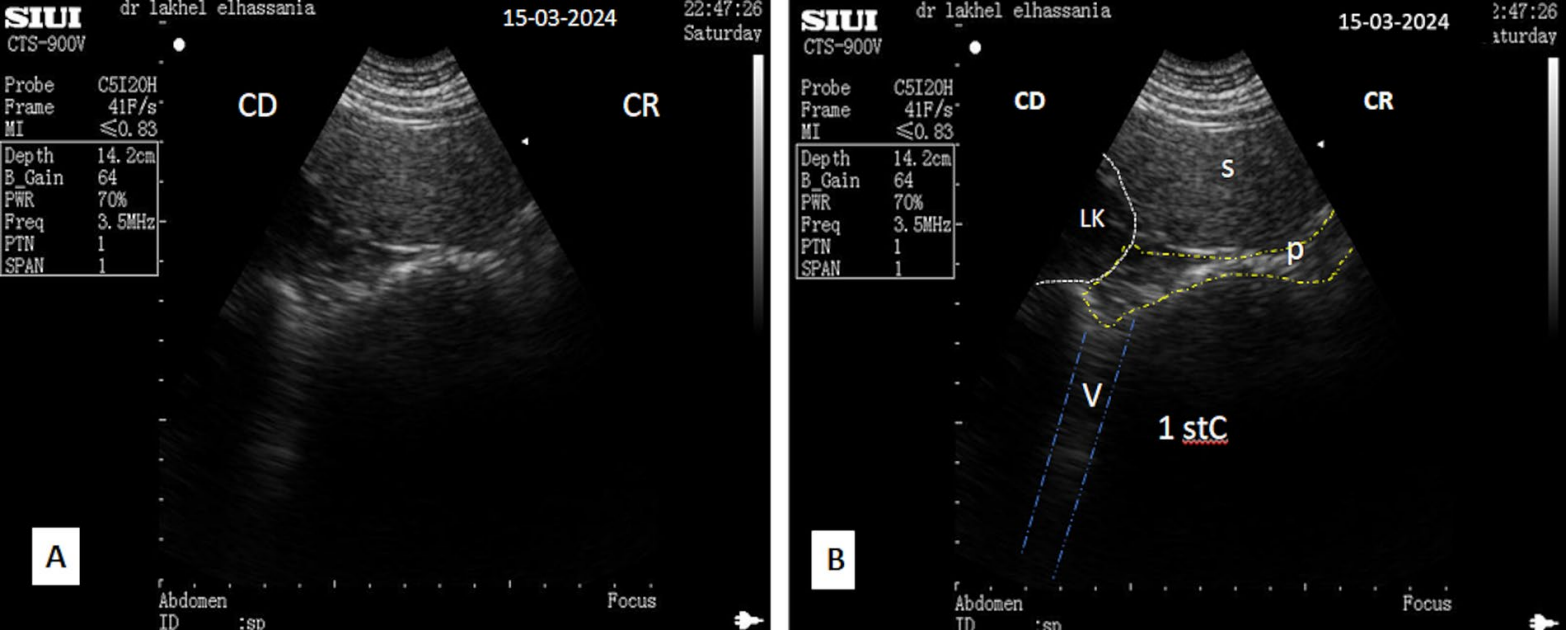
**(A)** Transverse scan of the left lobe of the pancreas ventrally to the anterior extremity of the spleen under second transverse process of the lumbar vertebra in the left flank in the contraction phase and (**B**, identification of structures on the ultrasound image); S, spleen; P, pancreas (yellow dotted line); LK, left kidney; 1st C, (first stomach compartment); V, vibration artifact or tail artifact (blue dotted line); CR, cranial; CD, caudal.

Ultrasound examination of non-fasted dromedaries showed acoustic shadows from solid food boluses in the duodenal ampulla, obscuring the pancreatic parenchyma (Figure 14). In contrast, food deprivation for 12 h eliminates this phenomenon (Figure 9). However, the thickness of the pancreatic parenchyma was reduced during the contraction phase of digestion in all animals, even after fasting (Figures 6A, 9A). Additionally, a comet tail artifact from the contractions of the distended rumen was observed above the left lobe, further complicating the interpretation of the ultrasonographic patterns (Figure 15).

## 4 Discussion

In the camel, ultrasonography has not yet been used as a basic diagnostic tool for abdominal viscera disorders, particularly those of the pancreas. In the present study, ultrasonography was used to explore the pancreas in healthy camels, combined with a detailed inspection of camel carcasses to enable direct assessment of the conformation and topography of the organ.

The results of the macroscopic anatomy and topographic investigation of the pancreas are consistent with previous findings in this species (12–16).

Furthermore, the pancreas differs from that of the ox by the development of the left lobe and the reduction in the right lobe as well as by the absence of the accessory pancreatic duct (14).

Despite its deep position, the pancreas is closely related to adjacent organs, fitting within the spaces between them (17). Ultrasound examination can effectively visualize the pancreas through various acoustic windows. The acquired anatomical observations from slaughtered animal carcasses provide valuable insights that can be reflected in the ultrasound images.

The ultrasonographic evaluation and measurement of the pancreas in young and adult camels were primarily conducted using the SIUI CTS-900 V ultrasound scanner, which demonstrated effective imaging capabilities for the pancreas. Various transducers were assessed, but the 3.5 MHz micro-convex probe was retained for its optimal ease of use, particularly in intercostal spaces. However, as the SIUI CTS-900 V ultrasound scanner does not incorporate Doppler functionality, a Samsung MH70A Doppler ultrasound scanner, a 3.5-MHz micro-convex probe, was also used for ultrasound examination in four females to visualize and assess the associated vessels.

The pancreatic body parenchyma was visualized by positioning the probe at the level of the portal hepatic incisure. This finding corroborates the descriptions provided by Mostafa et al. (12), as well as our anatomical observations on the carcasses of four young camels, confirming its position below the first and second lumbar vertebrae and behind the upper third of the 12^th^ rib. Mostafa et al. (18) and Barone (17) also describe the accessory lobe surrounding the portal vein, forming the pancreatic annulus. This structure appears as an anechoic pattern in ultrasonographic imaging and is confirmed by color Doppler ultrasonography. However, distinguishing the accessory lobe as a separate part remains a challenge.

The diameter of the portal vein observed in the body lobe is higher in adults but similar for both sexes. In transversal scans, it averages 1.8 ± 0.19 cm in young camels and 3.14 ± 0.18 cm in adults.

Furthermore, in camels, the ultrasonographic pattern of the pancreatic body appears hyperechoic, elongated, and irregular in shape, whereas imaging in cows reveals a triangular shape, as noted by Pusterla and Braun (19) and confirmed by Tharwat et al. (20); this triangular structure is related to liver, portal vein, right kidney, and duodenum. In cows, the ultrasonographic pattern of the body lobe is either isoechoic or slightly more echogenic than the reference ultrasonographic pattern of the liver.

The ultrasonography of the pancreatic right lobe of the camel revealed variations in shape and thickness across three acoustic windows. The parenchyma appeared elongated or trapezoid in the 11th intercostal space, triangular to prism-like in the 10th, and irregular or band-like in the 9th. It appears close to the duodenal ampulla in the 10th and 11th intercostal spaces, but it is near the abomasum and first stomach compartment in the 9th intercostal space. In contrast, according to Pusterla and Braun (19), the right lobe of the pancreas, in cows, is triangular and generally isoechoic or slightly more echogenic.

The thickness of the pancreatic parenchyma of the right lobe differed across the three acoustic windows; the lowest measurements were obtained in the 9th intercostal space, whereas the highest were in the 10th and a slightly reduced in the 11th. In all windows, the thickness was consistently greater in adults than in young camels. In contrast, the portal vein showed gradual widening across the windows.

Similar to cows (19) and dogs (21), the pancreatic ducts are visible in camels. In particular, the major pancreatic duct is present within the right lobe, as seen in cows, but is very clear in ultrasonographic patterns when scanned in the middle third of the 11th intercostal space (Figure 4). The mean diameter observed was 0.67 ± 0.05 cm (n = 14) for young camels and 0.79 ± 0.05 cm (n = 8) for adult camels, which is consistent with the macroscopic structure noted by Mostafa et al. (12). However, the accessory pancreatic duct is absent, which aligns with anatomical data.

The left lobe was successfully visualized by placing the probe in the left flank, specifically in the region defined cranially by the last rib, caudally by the left kidney, ventrally by the first stomach compartment (rumen), and dorsally by the spleen, which corroborates the description of Mostafa et al. (12). In contrast, the left lobe of the pancreas is not visible in cows, according to Tharwat et al. [19] and Pusterla and Braun (19).

The measurements of the pancreatic parenchyma were conducted through the intercostal spaces using both transversal and longitudinal scanning planes. The probe was oriented in multiple directions to ensure a comprehensive examination of the entire parenchyma, covering the different lobes of the pancreas, which sometimes allows measurements of length, width, and thickness. Imprecision may arise from the irregularity of the organ and its complex anatomical location. Therefore, specific measurements may not necessarily reflect the accurate macroscopic dimensions of the pancreas.

According to the macroscopic study of the pancreas of 14 young camels slaughtered at Sidi Bibi abattoir, the weight of the organ is nearly identical in females and males, which is 113.7 ± 14.9 g. Adult camels were not slaughtered in this abattoir, but according to Mostafa et al. (12), the weight of the pancreas can vary between 300 and 500 g. This finding explains the greater thickness of the pancreatic parenchyma of the body in the adults, measuring 4.05 ± 0.34 cm, and 3.60 ± 0.26 cm in young camels. Regarding the left lobe, it measured 2.32 ± 0.31 cm in adults and 3.08 ± 0.52 cm in young camels. Additionally, in the 10th intercostal space, the thickness of the right lobe was 5.9 ± 0.25 cm in the adult camels and 4.40 ± 0.20 cm in young camels.

Several pancreatic lesions have been described in cattle; in particular, pancreatic atrophy is associated with nutritional deficiencies, pancreatic lithiasis, and tumors (22). Because pancreatic diseases often elude clinical animal evaluation, veterinarians must rely on diagnostic tools that complement the patient’s history, clinical signs, and laboratory results, such as ultrasonography (19).

For example, in Denmark, cases of pancreatic calculi have been observed in slaughtered cattle over a 3-month period, with a frequency of 0.43% (23). Using ultrasound technology has been proven to be particularly valuable in diagnosing pancreatic calculi in cows exhibiting non-specific symptoms such as fever, anorexia, and weight loss. Ultrasonographic examination of the pancreas showed a distortion of its reference triangular shape, with a tendency toward a hypoechoic pattern and the presence of pancreatic stones accompanied by distal acoustic shadowing (24). In addition, the ultrasonographic patterns of experimentally induced pancreatitis in cattle showed that patchy hypoechoic foci developed within the pancreatic parenchyma (22).

The pancreatic pseudocysts are another example. One or more circular cystic structures with more or less well-defined contours within the pancreas were observed during ultrasound examinations in dogs. These structures are often wall-less and contain hypoechoic or even anechoic content; these structures typically lack walls and may contain hypoechoic or anechoic content, sometimes with hyperechoic debris dispersed throughout (25).

In addition, in the case of pancreatic abscesses in dogs, the pancreas shows a mass effect, and its echogenicity is variable: a hyperechoic mass with hypoechoic areas corresponding to fluid accumulations can be observed (26).

Pancreatic abnormalities in camels have not yet been described by ultrasound technique. Furthermore, reference models for the normal ultrasonographic appearance of the pancreas are not available, making the present study particularly valuable by providing essential data for the ultrasound examination in this species. However, pancreatic abnormalities have been well-defined in other species, which may be used as a reference for ultrasound examinations of pancreatic lesions in dromedary camels while pending the implementation of such examinations.

Pancreatic lesions represent a subtle and often overlooked pathological case in animals. They manifest as progressive weight loss, affecting the fattening process, leading to reduced meat yield and significant economic losses for farmers. This case underscores the importance of incorporating ultrasonography, which may help in detecting pancreatic lesions and implementing appropriate treatment.

In addition to the challenges associated with ultrasonography in camels compared to other species, such as the need for experienced handlers for restraint, animal preparation time is also crucial. It includes meticulous shaving to ensure better contact between the abdominal wall and probe and generous gel application. The frequent agitation of camels can disrupt the ultrasound trial by displacing the organs and altering their stability. Therefore, light sedation or anesthesia may be necessary, as it helps slow down respiratory movements and ruminal contractions, which is beneficial for obtaining more explicit ultrasound images.

Ultrasound can be performed in either a standing or sitting position. Sometimes, the sitting position is the only option when the animal cannot be anesthetized or is too agitated, but it is essential to take into account the displacement of organs. It is recommended that the animal be deprived of food to prevent interference from ruminal overload. Movements caused by ruminal contractions and respiration can create artifacts and disrupt organ visualization. Consequently, synchronizing the examination with the respiratory phase may help optimize image quality.

Finally, a micro-convex probe is ideal for pancreatic ultrasonography. It facilitates exploration and a better visualization of the organ through narrow intercostal spaces with a frequency of 3.5 MHz reaching a minimum depth of 14 cm, especially as the organ is anatomically deep. The Samsung MH70 ultrasound scanner stands out for its superior resolution and advanced tools, including the Doppler function, which provides valuable information about vascularization. In contrast, the SIUI CTS-900 V ultrasound scanner is effective for routine examinations but is limited by its requirement for Doppler capability. Consequently, the choice of ultrasound machine will depend on the specific needs of the veterinarian and the available budget.

## 5 Conclusion

Ultrasonography is a reliable and non-invasive diagnostic modality for the assessment of the pancreas in various species, including the dromedary camel, despite the deep anatomical positioning of the organ. This study aimed to elucidate the ultrasonographic characteristics of the pancreatic tissue in dromedary camels, thereby advocating for its incorporation into routine veterinary practice. Establishing a reference database for normal pancreatic ultrasonographic patterns and values will enable veterinarians to identify abnormalities, facilitating early detection of pancreatic lesions, particularly in cases where clinical symptoms are non-specific.

Early diagnosis and prompt intervention in pancreatic disorders are crucial for mitigating potential complications, enhancing clinical outcomes, and ultimately reducing economic losses for camel husbandry. Given the increasing challenges posed by climate change, improving the health and productivity of camel livestock through advanced diagnostic techniques is imperative. The findings of this study underscore the significance of ultrasonography as a vital tool for the veterinary assessment of pancreatic health in dromedary camels, contributing to both animal welfare and agricultural sustainability.

## Data Availability

The original contributions presented in the study are included in the article/supplementary material, further inquiries can be directed to the corresponding authors.
